# Challenges of Recruiting Persons with Alzheimer’s Dementia (AD) for Genetic Studies in Selected African Countries on the African Dementia Consortium (AfDC)

**DOI:** 10.1002/alz.090911

**Published:** 2025-01-09

**Authors:** Olufisayo Oluyinka Elugbadebo, Temitope Hannah Farombi, Mayowa Ogunronbi, John‐Paul Umuojine, Yared Z Zewde, Christine W. Musyimi, Judith Boshe, Kamada Lwere, David Ndetei, Albert Kwaku Akpalu, Fred Stephen Sarfo, Alfred Kongnyu NJAMNSHI, Patrice L. Whitehead, Larry D. Adams, Inna Logvinsky, Jovita D. Inciute, Brain Kunkle, Anthony J. Griswold, Jacob L McCauley, Jeffery M. Vance, Margaret A Pericak‐Vance, Oyedunni Arulogun, Olusegun Baiyewu, Adesola Ogunniyi, Rufus O. Akinyemi, Africa Dementia Consortum

**Affiliations:** ^1^ College of Medicine, University of Ibadan, Ibadan Nigeria; ^2^ University College Hospital, Ibadan Nigeria; ^3^ Institute for Advanced Medical Research and Training, College of Medicine, University of Ibadan, Ibadan, Oyo State Nigeria; ^4^ Komfo Anokye Teaching Hospital, Kumasi Ghana; ^5^ Global Brain Health Institute, University of California, San Francisco, CA USA; ^6^ Africa Mental Health Research and Training Foundation, Nairobi Kenya; ^7^ Kilimanjaro Christian Medical University College, Moshi Tanzania, United Republic of; ^8^ Makerere University, Kampala Uganda; ^9^ University of Nairobi, Nairobi Kenya; ^10^ University of Ghana, Accra Ghana; ^11^ Brain Research Africa Initiative, Yaounde, Centre Cameroon; ^12^ John P. Hussman Institute for Human Genomics, University of Miami Miller School of Medicine, Miami, FL, USA, Miami, FL USA; ^13^ University of Miami, Miami, FL USA; ^14^ John P. Hussman Institute for Human Genomics, University of Miami Miller School of Medicine, Miami, FL USA; ^15^ University of Miami, Miller School of Medicine, Miami, FL USA; ^16^ University of Miami Miller School of Medicine, Miami, FL USA; ^17^ 1501 NW 10th Avenue, Miami, FL USA; ^18^ University of Ibadan, Ibadan, Oyo Nigeria; ^19^ College of Medicine, University of Ibadan, Ibadan, Oyo State Nigeria; ^20^ College of Medicine, University of Ibadan, Ibadan, Oyo Nigeria; ^21^ Neuroscience and Aging Research Unit, Institute of Advanced Medical Research and Training, College of Medicine, University of Ibadan, Ibadan Nigeria

## Abstract

**Background:**

Understanding the genetic underpinnings of Alzheimer’s disease is crucial for advancing research and developing targeted interventions. Genomic research in dementia in Africa is of utmost importance based on recent reports from studies in African Americans that African ancestral gene is associated with lower risk effect for developing AD. However, dementia related genetic study is an evolving research in sub‐Saharan Africa with peculiar challenges influencing participant recruitment. This study sought to identify the key challenges of the recruitment process into dementia‐ related genetic studies and how these were mitigated in the READD‐ADSP Africa and Origins of AD in African ancestry genetic studies.

**Method:**

A narrative qualitative research design using in‐depth open‐ended interviews explored the challenges of recruiting participants and how these were managed among the key stakeholders, including, healthcare professionals, and researchers involved in the recruitment process from nine African countries participating in the AfDC. An inductive thematic analysis was applied to systematically code and analyze the data.

**Result:**

Eighteen stakeholders from 7 African country members (Nigeria, Ghana, Kenya, Tanzania, Uganda, Cameroon, and Ethiopia) of the AfDC were interviewed. Similar challenges were observed across most African Countries which includes non‐existing national dementia registry, a common challenge in the recruitment process. Other challenges identified were poorly populated local or hospital‐based dementia registry, language diversity, myths around blood collection, family dynamics, stigma, logistics, unmet expectations concerning incentives, fewer older controls and data privacy. Leveraging on previous research programmes, existing community engagement activities and client‐doctor relationship assisted in addressing these challenges

**Conclusion:**

There are some unique challenges with recruitment of participants into genetic studies in Africa. Educating the populace on the need for genetic studies is key to addressing some of these challenges. Community engagement programs specifically targeted at improving knowledge about dementia are currently being deployed across these countries. Adopting culturally‐sensitive recruitment strategies, strengthening community engagement and advocacy for well populated dementia registry for AfDC could further help address these obstacles and improve recruitment of participants in genetic studies.

